# High-LD SNP markers exhibiting pleiotropic effects on salt tolerance at germination and seedlings stages in spring wheat

**DOI:** 10.1007/s11103-022-01248-x

**Published:** 2022-02-25

**Authors:** Nouran M. Hasseb, Ahmed Sallam, Mohamed A. Karam, Liangliang Gao, Richard R. C. Wang, Yasser S. Moursi

**Affiliations:** 1 Department of Botany, Faculty of Science, Fayoum University, Fayoum, 63514 Egypt; 2 Department of Genetics, Faculty of Agriculture, Assiut University, Assiut, 71526 Egypt; 3 Department of Plant Pathology and Wheat Genetics Resource Center, Kansas State Univ, Manhattan, KS 66502 USA; 4 Agricultural Genomics Institute at Shenzhen, Chinese Academy of Agricultural Sciences, Shenzhen, Buxin Road 97, Dapeng-District, Shenzhen, 518120 Guangdong China; 5 USDA-ARS Forage and Range Research Lab, Utah State University, Logan, UT 84322-6300 USA

**Keywords:** Salt stress, Wheat, Candidate gene, QTL validation, GWAS

## Abstract

**Key message:**

Salt tolerance at germination and seedling growth stages was investigated. GWAS revealed nine genomic regions with pleiotropic effects on salt tolerance. Salt tolerant genotypes were identified for future breeding program.

**Abstract:**

With 20% of the irrigated land worldwide affected by it, salinity is a serious threat to plant development and crop production. While wheat is the most stable food source worldwide, it has been classified as moderately tolerant to salinity. In several crop plants; such as barley, maize and rice, it has been shown that salinity tolerance at seed germination and seedling establishment is under polygenic control. As yield was the ultimate goal of breeders and geneticists, less attention has been paid to understanding the genetic architecture of salt tolerance at early stages. Thus, the genetic control of salt tolerance at these stages is poorly understood relative to the late stages. In the current study, 176 genotypes of spring wheat were tested for salinity tolerance at seed germination and seedling establishment. Genome-Wide Association Study (GWAS) has been used to identify the genomic regions/genes conferring salt tolerance at seed germination and seedling establishment. Salinity stress negatively impacted all germination and seedling development parameters. A set of 137 SNPs showed significant association with the traits of interest. Across the whole genome, 33 regions showed high linkage disequilibrium (LD). These high LD regions harbored 15 SNPs with pleiotropic effect (i.e. SNPs that control more than one trait). Nine genes belonging to different functional groups were found to be associated with the pleiotropic SNPs. Noteworthy, chromosome 2B harbored the gene *TraesCS2B02G135900* that acts as a potassium transporter. Remarkably, one SNP marker, reported in an early study, associated with salt tolerance was validated in this study. Our findings represent potential targets of genetic manipulation to understand and improve salinity tolerance in wheat.

**Supplementary Information:**

The online version contains supplementary material available at 10.1007/s11103-022-01248-x.

## Introduction

Worldwide, 45 million hectares (~ 20%) of the irrigated land and 32 million ha of dry land (2%) are salt-affected. In addition, 1.2 million have become salt-affected annually (FAO/AGL 2018). To meet the need for continued human population growth, more than 70% increase in global food production is required by 2050 (Tester et al. 2010). With the effect of global climate changes, understanding the plant salinity tolerance mechanisms is critical to sustaining wheat yield. Salinity causes osmotic stress and/or ion toxicity, delays or inhibits seed germination, and curtails plant development. With the progressive expansion of salinity-affected soils, salinity becomes a noxious stress that hinders crop production (Munns and Tester 2008). Among cereals, common wheat (*Triticum aestivum L.*) is the most widely cultivated cereal crop. It shows a high sensitivity to soil salinization relative to its halophytic relative tall wheatgrass (*Thinopyrum ponticum* (Podp.) Barkworth & D.R. Dewey) which can withstand high levels of soil salinity (Zhao et al. 2016). Improving salinity tolerance in wheat is a must to sustain grain yield in production under saline soil condition, and to meet the rapidly growing global food demand (Borjigin et al. 2020).

Seed germination and seedling establishment are critical stages of plant development (Bewley 1997). However, seed germination represents the shortest period in plant development; it is a multistage phenomenon with high phenotypic and genotypic variation under salinity (Song and Xing 2010; El-Hendawy et al. 2019). Francois et al. (1986) found that wheat is more sensitive to salt stress at germination than at the three-leaf stage. Conventional agronomic and engineering approaches did not achieve the targeted improvement in salt tolerance, thus the best approach to increase salt tolerance is to develop more tolerant genotypes (Munns and Gilliham 2015). Additionally, the selection of salt-tolerant genotypes based on morphological or yield-trait attributes might be non-predicative. In wheat, Royo and Abió (2003) found a 30–40% lower salt tolerance than those estimated by Maas and Hoffman (1997). These limitations can be surpassed by using the most recent molecular approaches such as marker-assisted selection (MAS). Genetic diversity for salt tolerance in bread wheat is limited, for example, one Indian landrace (Kharcia 65) played an instrumental role in salt-tolerant genotypes production, where the cultivars KRL1-4 and KRL 19 were introduced later (Oyiga et al. 2018). Many studies investigated the genetic diversity for salt tolerance in wheat at various growth stages (Rahnama and Munns 2011; Probert et al. 1998; Lindsay et al. 2004b; El-Hendawy et al. 2009; Oyiga et al. 2018), making a great opportunity to improve salt tolerance in wheat.

Capturing the genetic variation for salt tolerance in wheat using the biparental quantitative trait loci (QTL) mapping was done successfully at seed germination and seedling stage. The *Nax1* locus on chromosome 2AL is responsible for Na^+^ exclusion (Lindsay et al. 2004a). Similarly, another QTL for Na^+^ exclusion on 7AS was detected in two double haploid populations (Edwards et al. 2008). Ma et al. (2007) mapped 47 and 37 QTLs for salinity tolerance at seed germination and seedling stage, respectively.

Genc et al. (2010) found that the locus for detected Na^+^ exclusion on 2A has a positive effect (10% increase) on seedling biomass. Furthermore, they found two QTLs for Na^+^ exclusion co-localized with seedling biomass QTLs (on 2A and 6A). Despite the successful identification of QTL for salinity tolerance at seed germination and seedling stage, it is not easy to precisely detect the candidate genes/loci for salt tolerance in bread wheat using QTL mapping. This is attributed to the complex nature of the salinity tolerance, as well as the uncoupling of the two main phases of salinity (osmotic and ion-toxicity) is very difficult (Munns 2002). Moreover, QTL mapping requires the time-consuming production of a suitable mapping population, which might still result in the low resolution of QTL detection, in conjugation with identifying a limited number of alleles at the given loci (Flint-Garcia et al. 2003).

Genome-wide association study (GWAS) represents a powerful alternative approach to detect the significant marker-trait association (MTAs), via employing the historical recombination and mutation events in a given species. GWAS has been proven to be a powerful tool to disentangle the genetic architecture of the complex biotic and abiotic stresses in various plant species (Oyiga et al. 2018). Recently, GWAS has been successfully used to identify the QTL governing salt tolerance at seed germination in various plant species including wheat (Oyiga et al. 2018; Beyer et al. 2019; Yu et al. 2020), rice, (Yu et al. 2018; Naveed et al. 2018), barley (Thabet et al. 2020; Mwando et al. 2020), sesame (Li et al. 2018), soybean(Kan et al. 2015; Do et al. 2019) cotton (Sun et al. 2018), and alfalfa (Yu et al. 2016a). Moreover, it has been more often used to identify QTL for seed quality and grain yield attributes under non-stressful conditions (Jiang et al. 2017; Guo et al. 2017; Sun et al. 2017), as well as under salinity stress (Hussain et al. 2017; Oyiga et al. 2018).

The objectives of the current study are (1) to estimate the salinity tolerance variation at seed germination and seedling establishment in a diverse set of bread wheat representing 176 genotypes, (2) to identify the significant marker-trait associations that regulate seed germination and seedling establishment under salinity stress.

## Material and methods

### Plant material

A diverse panel of bread wheat including 176 genotypes from 22 different countries was tested for seed germination and seedling establishment under salinity stress. The full description of the panel is illustrated in Table S1. These genotypes were selected for this study based on their high adaptability to the normal Egyptian conditions (Ahmed Sallam, personal communication).

### Germination and seedling development experiment layout

All genotypes were tested for salinity tolerance during seed germination and seedling establishment in a randomized complete block design (RCBD) with three replications. Twenty seeds from each genotype were washed with water and sterilized in 1% Sodium hypochlorite (NaOCl) for 10 min, thereafter, rewashed three times with deionized distilled water. The twenty seeds were placed in Petri dishes on two layers of filter papers (Whatman, No 1) moistened by 10 ml of the corresponding solutions; (0 mM NaCl; control) and (175 mM NaCl; salinity stress). The Petri dishes were incubated at 20 °C in the darkness. To maintain the initial salt stress, the NaCl solution was replaced every second day until the end of the experiment. The seed was considered germinated when the radicle became 2 mm long. The germination has been scored every 24 h up to 10 days. At the end of the 10th day, several germination- and seedling-related traits were calculated. For shoot length (SL) and root length (RL) measurements, from each genotype, ten seeds were grown in a rolling paper following Hetz et al. (1996). Thereafter, the rolling papers were placed in 1L beakers half-filled with the corresponding solutions (0 mM-NaCl, control and 175 mM NaCl, salinity stress). To maintain the initial salinity strength as well as the volumes of the initial solutions, the solutions were refreshed every second day until the end of the experiment. After 12 days, the experiment was terminated and SL and RL (in cm) were manually measured using a scaled ruler. The mean values over the three replications were calculated and have been used for GWAS analysis.

Germination perecentage (G%) was calculated using the formula below.

$$\scriptsize {\text{G}}\% \, = \,{\text{Number of germinated seeds at the 10}}{\text{th day}}/{\text{Total number of seeds}}\, \times \,{\text{100.}}$$

Germination Pace (GP) was calculated using the formula below

$$GP = \frac{N}{{\sum \left( {n \times g} \right)}} \times 100$$

where N is the number of germinated seeds at the end of the experiment, n is the number of newly germinated seeds at a certain day g, g = (1, 2, 3,….).

Root/shoot ratio (RSR) was calculated as the ratio of the root length to the shoot length.

Number of Roots (NoR) was counted visually as the total number of roots on the 10th day.

Fresh Weight (FW) was recorded in (g) using a sensitive balance (Sartorius AC 1215, Germany).

Salt Tolerance Indices (STIs) were calculated for all estimated traits except SRR and NoR, using the equation below

$$STI = \frac{Trait \,value \,under \,salinity}{{Trait \,value \,under\, control}} \times 100$$

The traits nomenclature under the different treatments includes the trait abbreviation followed by the sign C for control and the sign S for salinity.

### Genotyping and SNP developing

The genotypes of this study were among a set of 2152 genotypes which were genotyped with the Illumina’s iSelect 9K SNP array at the USDA-ARS Biosciences Research Laboratory in Fargo, ND (Cavanagh et al. 2013). A set of 6883 SNPs for the 176 genotypes, used in this study, were obtained from Gao et al. (2017).

In the current study, the 6883 markers were filtered based on minor allele frequency (0.05) and missing data percentage (20%). As a result, 6141 markers were retained and used for GWAS. A mixed linear model (MLM) + kinship was used to identify the maker-trait association using TASSEL v5.2.40 (Bradbury et al. 2007). The population structure was tested using principal component analysis (PCA) based on genetic distance using TASSEL. Marker-trait association was detected at a significant threshold of 0.001 p value (Bradbury et al. 2007). The allele effects of target SNP and phenotypic variation explained by marker (R^2^) were estimated by TASSEL software. Linkage disequilibrium (*r*^*2*^*)* was calculated for only significant SNPs located on the same chromosome using TASSEL software. The marker-trait association was detected at p < 0.001. Also, the multiple testing using false discovery rate (FDR) with an α level of 0.20 was used in GWAS according to He and Lin (2011), Juliana et al. (2021), and Dolejsi et al. (2014)

Population structure among all genotypes in the population was investigated using principal component analysis (PCA) based on the genetic distance among the genotyping. All SNP markers were used in the PCA and the PS was performed using TASSEL software.

### Gene annotation and gene expression

The gene annotation has been conducted based on a BLAST-based approach. The sequence of the SNP marker has been blasted against the functional annotation provided by the International Wheat Genome Sequencing Consortium (IWGSC) wheat assembly from EnsemblPlants release-51 (Howe et al. 2020). (https://plants.ensembl.org/Triticum_aestivum/Info/Index?db=core).

The gene expression values were calculated as the transcript per million (tpm), for each gene in root and shoot under control and abiotic stress of previously mapped RNA-seq samples from the RefSeqv1.1 assembly (Ramírez-González et al. 2018) (www.wheatexpression.com).

### Data analysis

The analysis of variance (ANOVA) was calculated for all estimated traits under control and salt stress by PLABSTAT software [43] and R package [44] using the following statistical model.

$$Y_{ijk} = \, \mu \, + \, g_{i} + \, r_{j} + \, t_{k} + \, t_{ik} + \, tgr_{ijk}$$

where Y_ijk_ is the observation of genotype i in replication j in treatment (normal vs salt stress) k, μ is the general mean; g_i_, r_j_, t_k_ are the main effects of genotypes, replications, and treatments, respectively. t_ik_ is genotype × treatment interaction. *tgr*_*ijk*_ is genotype × replications × treatment interaction (error).

Broad-sense heritability (H^2^) was estimated by PLABSTAT using the following equation

$${H}^{2}= \frac{{\sigma }_{G}^{2}}{{\sigma }_{G}^{2}+(\frac{{\sigma }_{GR}^{2}}{r})}$$

where G refers to genotypes and r refers to replications.

Phenotypic correlation analysis was estimated by PLABSTAT. Correlation coefficients ranging from 0 to 39 were considered low, 0.40–0.60 were moderate, and above 0.60 were high.

## Results

### Phenotypic characterization and evaluation

**Fig. 1:**
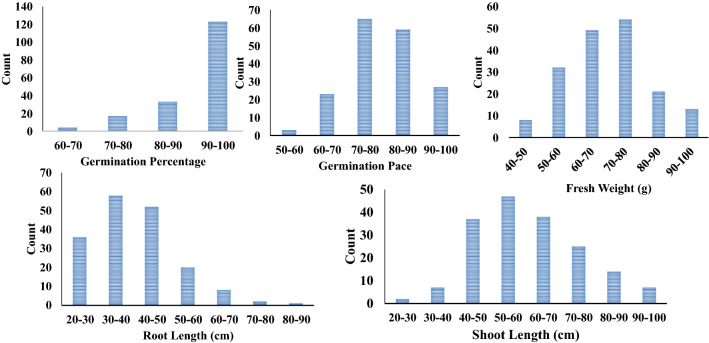
Histogram shows the distribution salt tolerance index (STI) of traits during seed germination and seedling development in wheat under control (0-NaCl) and salt stress (175-NaCl)

Under the two treatments (0 mM NaCl, control and 175 mM NaCl, salinity stress), a diverse panel consisting of 176 genotypes were characterized for salinity tolerance at seed germination and seedling establishment. Six traits were measured to estimate the genotypic variation for salinity tolerance. Besides, the Salt Tolerance Indices (STIs) were estimates of reduction that resulted from salinity stress. All measured traits; Germination rate (G%), germination Pace (GP), shoot length (SL), root length (RL), root/shoot ratio (RSR), number of roots (NoR), and fresh weight (FW) were negatively affected by salinity stress. Table 1 shows that all traits were reduced significantly under salinity stress relative to control. The performance of each genotype in all traits under both conditions is presented in supplementary Table 2. The lowest reductions were detected for G% and GP with salt tolerance indices (STIs) of 91 and 79.63 %, respectively. The highest reductions were detected for SL and RL with STIs of 60 and 40.28 %, respectively (Table 1). These results showed that salinity inhibited shoot and root elongation more than inhibiting seed germination. All traits exhibited normal distributions under control and salinity except G% (Supplementary Fig. 1). Similarly, the corresponding STIs of all traits showed normal distributions, except for G% (Fig. 1).

**Table 1:** Ranges, means and analysis of variance (ANOVA) for all traits scored on wheat under control (0 mM-NaCl) and salinity (175 mM-NaCl)

Ranges and means of traits						
	Control			Salinity		
	Min	Max	Mean	Min	Max	Mean
Germination percentage (G%)	75.00	100.00	98.45	60.00	100.00	90.01
Germination pace (GP)	45.00	77.00	58.60	36.36	65.97	46.81
Shoot length (SL) (cm)	6.20	18.50	11.38	3.00	10.10	6.71
Root length (RL) (cm)	8.50	23.00	15.94	3.50	10.80	6.26
Root-shoot length ration (RSR)	0.71	2.54	1.44	0.45	2.10	0.96
Number of roots (NoR)	3.00	8.00	4.65	3.00	6.00	4.90
Fresh weight (FW) (gm)	2.10	7.80	5.44	1.10	5.40	3.78

Ranges and means of salt tolerance index	Min	Max	Mean
Germination Percentage Salt Tolerance Index (G%-STI) (%)	60.00	100.00	91.00
Germination Pace Salt Tolerance Index (GP-STI) (%)	56.00	100.00	79.63
Shoot Length Salt Tolerance Index (SL_STI) (%)	27.27	100.00	60.74
Root Length Salt Tolerance Index (RL_STI) (%)	20.93	95.29	40.28
Fresh Weight Salt Tolerance Index (FW_STI) (%)	37.39	100.00	70.01

Analysis of variance (ANOVA)						
Source of variance	G%	GP	SL	RL	RSR	FW
Treatments	152.74**	471.54**	779.08**	2111.57**	247.73**	13.29**
Replications	0.14	0.32	0.03	0.79	0.69	0.17
Genotypes	87.49**	50.27**	59.33**	55.54**	39.55**	9.56**
Treatment × Genotype	56.38**	42.06**	42.04**	58.91**	41.54**	6.03**
Heritability	98.86	98.01	98.31	98.2	97.47	89.54

Min stands for minimum, Max for Maximum and STI for Salt Tolerance Index

*,**, ***Stand for significance levels P ≤ 0.05, 0.01 and 0.001, respectively

The analysis of variance (ANOVA) revealed high genetic differences among genotypes for all traits, respectively (Table 1). The differences due to the replications effect were not significant. High significant differences were found between treatments (salt vs control). The interaction between genotypes and treatments was highly significant. All traits had high heritability estimates (H^2^) ranging from 89.54 (FW) to 98.86 (G%).

### Correlation analysis

**Fig. 2:**
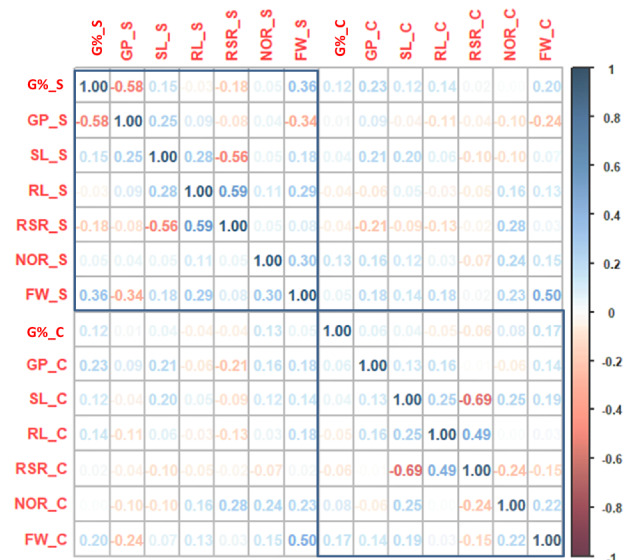
Phenotypic correlations among all traits scored under both conditions

Pearson's correlation analysis was conducted for all traits. Overall, the seed germination-related traits; G% and GP were less correlated than the seedling-related traits; SL, RL, RSR, and FW. Under control, mostly the positive and negative significant correlations were low to moderate. The highest negative correlation was detected between SLC and RSRC with r = − 0.69***, whilst the highest positive and significant correlation was observed between RLC and RSRC with r = 0.49** (Fig. 2). Under salt stress, G%S and GPS showed a highly significant negative correlation (r = − 0.57**). Also, a positive and significant correlation between RLS and RSRS increased (r = 0.59***), whilst the negative correlation between SLS and RSRS decreased (r =  − 0.56**). FWS showed significant positive correlations with all traits except with GPS (Fig. 2).

**Fig. 3:**
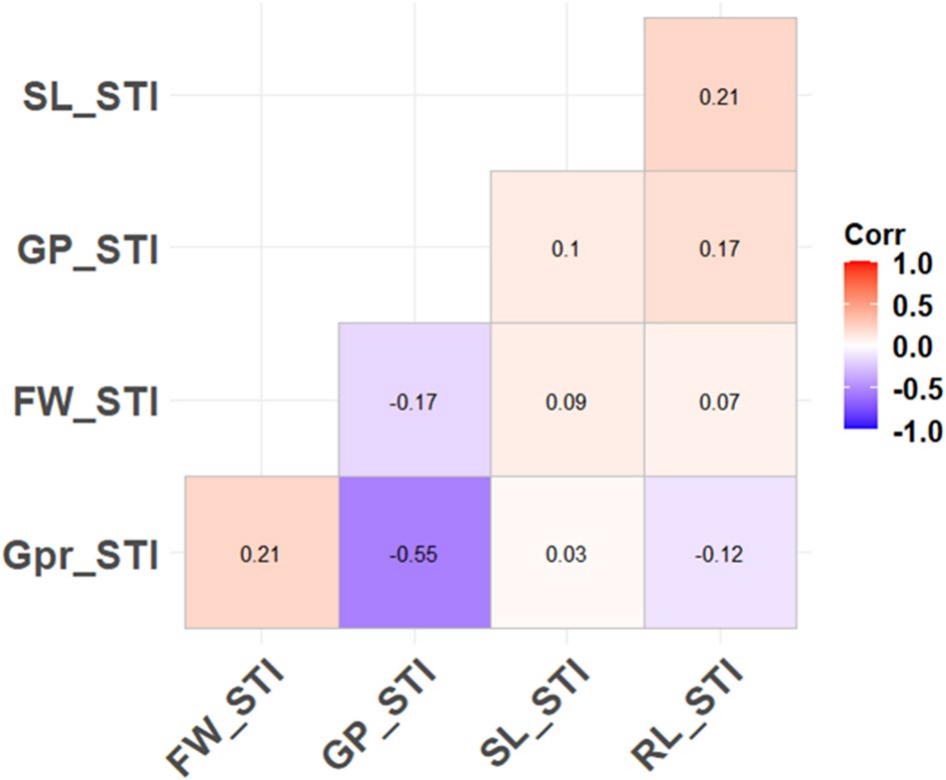
Phenotypic correlation among the selection indices

For salt tolerance indices, GP_STI had a negative and significant correlation with G%_STI (r =  − 0.55**)  (Fig. 3). No or weak significant correlations were found among the other indices.

### Phenotypic selection for salt-tolerant genotypes

The five selection indices were used to identify the most salt-tolerant as well as susceptible genotypes (Table 2). Firstly, all genotypes were sorted based on their tolerance in each index. Secondly, the most 10 salt-tolerant genotypes in each index were selected. Thirdly, the genotype was finally selected if it was among the 10 salt tolerant genotypes in at least two indices. As a result, a set of seven genotypes were considered tolerant to salt stress. These seven genotypes were from Egypt, Afghanistan, Algeria, Australia, Morocco, and Oman. All tolerant genotypes were among the best 10 genotypes in two different indices except PI 542666 (Afghanistan) and PI 525241 (Morocco) which were among the best genotypes in three indices. No tolerant genotype was found to be among the best 10 genotypes in all indices.

**Table 2:** The most salt tolerant genotypes (T) and susceptible genotypes (S) in more than one trait

Genotypes	Country	Characterization	G%_STI	SL_STI	RL_STI	FW_STI	No. of traits
PI 220127	Afghanistan	T	** × **			** × **	2
PI 542666	Algeria	T	** × **	** × **			2
PI 201414	Australia	T		** × **		** × **	2
PI 525241	Morocco	T	** × **		** × **	** × **	3
PI525221	Morocco	T	** × **			** × **	2
PI532249	Oman	T	** × **			** × **	2
PI574346	Saudi Arabia	S			** × **	** × **	2
PI599988	USA	S	** × **	** × **			2
Sohag-5	Egypt	S	** × **	** × **			2
Giza-156	Egypt	S		** × **	** × **		2
PI438961	Kazakhstan	S		** × **	** × **		2
PI525295	Morocco	S	** × **			** × **	2

^x^ Refers to the presence of the genotypes among the most 10 tolerance or susceptible genotypes for the respective trait

Likewise, the 10 susceptible genotypes were determined using the aforementioned approach. As a result, six genotypes from Saudi Arabia, the USA, Egypt, Kazakhstan, and Morocco were considered susceptible to salt stress. All the susceptible genotypes were among the most susceptible genotypes in two indices (Table 2).

### Genome-wide association study for salt tolerance at germination and seedling stages

**Fig. 4:**
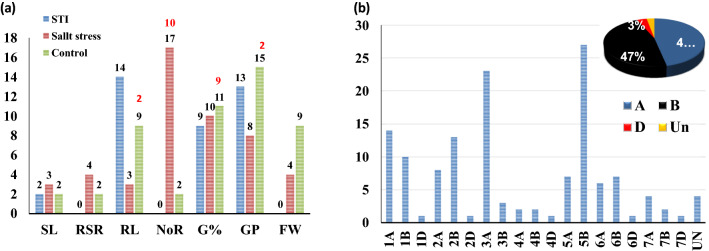
**a** Number of QTLs detected under salt stress and normal conditions, **b** number of QTLs per chromosome and in each genome. The black number stands for QTLs detected at p < 0.001, while red numbers refer to QTLs detected at p < 0.2 FDR

**Fig. 5:**
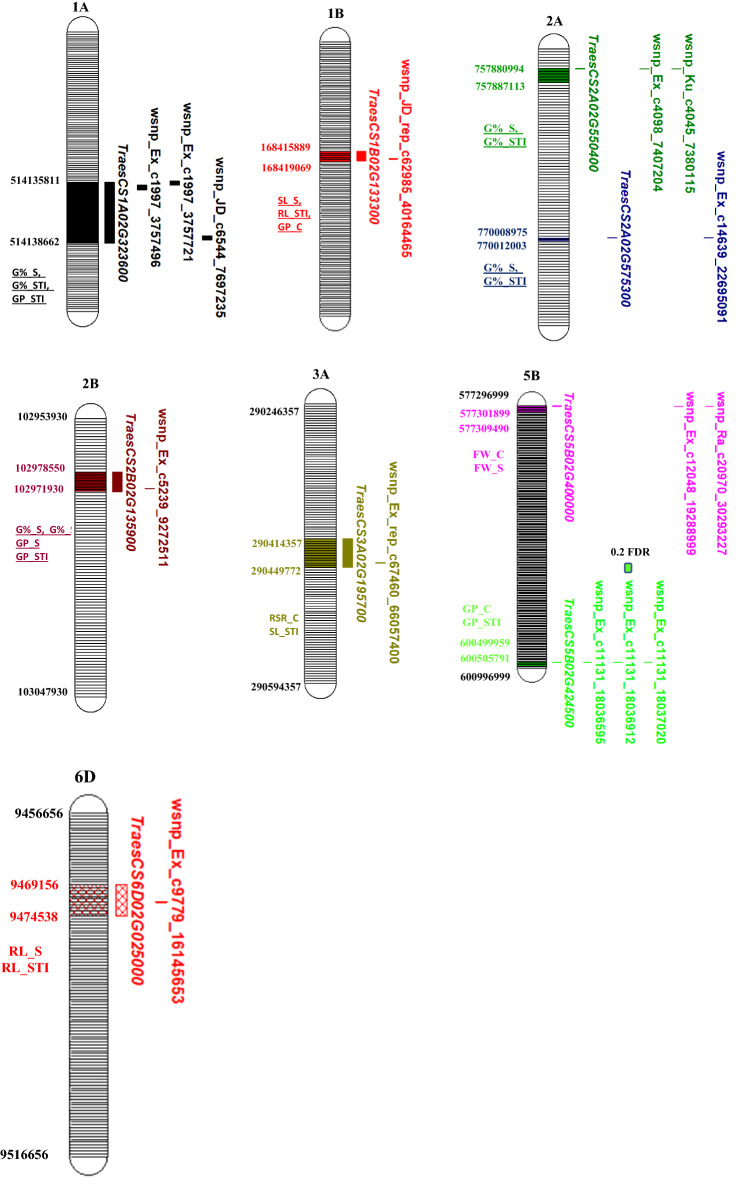
Physical positions (bp) of the common SNP markers and their gene models on the wheat chromosomes

A set of 6883 SNP markers was used to identify alleles associated with salt tolerant traits scored in this study. These SNPs were distrusted on all wheat chromosomes. Out of the 6883 SNPs, 604 were assigned as unknown chromosomal positions. The analysis of population structure using the PCA approach divided the genotypes into very large and small groups, indicating the presence of relatedness in the population (Supplementary Fig. 2). The GWAS revealed 137 and 23 significant markers associated with traits under control and salt stress conditions at significant levels of p < 0.001 and 0.2 FDR, respectively. The q-q plots for all traits are presented in Supplementary Fig. 3. The number of QTLs for each trait under both conditions is illustrated in Fig. 4a. Approximately the same number of QTL was detected under control (50) and salt stress (49), while 38 QTLs were associated with the five indices at p > 0.001 (Fig. 4a). At 0.2 FDR, 10 QTL were detected for the number of roots under salt stress, while 13 QTLs were found for G% (nine), RL (two) and GP (two) (Fig. 4a). Under control conditions, two QTLs were found for SL, NoR, and RSR, while 15 QTLs were found for GP. On the other hand, three QTLs for SL and RL were under salt stress, while 17 QTLs were found for NoR. For the five indices, two QTLs were detected for SL_STI, while 14 QTLs were found for RL. No QTLs were detected in STI_RSR, STI_NOR, and FW. On the chromosomal level, the number of QTL were distributed on all the 21 chromosomes extending from one QTL on 1D, 2D, 4D, 6D, and 7D, to 27 QTLs on 5B with the highest number of QTLs (Fig. 4b). On the genome level, genome A and B harbored the same number of QTLs (64 QTL) with 47% for each. Genome D had only 5 QTLs representing 3% of total QTLs. Only four QTLs were located in unknown chromosomal positions. The significant level of 0.2 FDR was used to detected markers with small effects (Fig. 5). Detecting markers with large and small effects are very important to understand the genetics of complicity traits such as salt tolerance (Sallam et al. 2016).

The summary of GWAS analysis is presented in Table 3, while a detailed GWAS result is represented in Supplementary Table 2. For FW, the number of detected SNPs under control (nine) was more than those detected under salt stress (four). Under control conditions, four major QTLs were found with R^2^ extended from 10.01 to 10.86%, while only two major QTLs were detected under salt stress (10.19 and 10.191%). Notably, SNP marker clusters (markers that were mapped in the same position) were observed on chromosome 2B (four) and 5B (two markers). All SNP markers associated with FW under salt stress were located on 5B with two markers in the same position. The target allele effect for each significantly associated with increased FW ranged from 0.52 to 0.79% and from 0.53 to 0.65% under control and salt stress, respectively.

**Table 3:** Summary of GWAS results for salt tolerance at seedling and germination stages, under control (C) and salinity (S)

Trait	treatment	Chromosome	P value	R^2^
Germination percentage	C	5B, 3A, 6B, 1B, 1A, 7A	0.000000098–0.000166	7.9–9.1%
	S	5A, 6A, 6D, 2B, UN	0.00018–0.00086	7.8–19.2%
Germination pace	C	4B, 3B, 5B, UN, 3A, 7D	0.000000018–0.000153	8.2–21.6%
	S	2A, 1A, 1D, 2B	0.00026–0.00097	7.9–10.1%
Fresh weight	C	2A, 2B, 4B, 5B	0.00012–0.00097	9.7–10.8%
	S	5B	0.00014–0.00098	8.0–10.4%
Number of roots	C	5A	0.00059	8.5%
	S	3A, 5B, 6B, 7A	0.001–0.000058	7.9–11.9%
Root length	C	7B. 6B. 2A. UN, 3A, 4A	0.000053–0.000164	8.0–11.8%
	S	6A, 7A, 5B	0.00014–0.00094	8.1–17.5%
Shoot length	C	6B	0.00046–0.00094	8–9%
	S	1B, 2B	0.00018–0.00088	7.8–9.8%
Root/shoot ration	C	3A, 1B	0.00014–0.00099	8.1–10.7%
	S	5B, 6A	0.00057–0.00082	8.2–8.6%
STI (G%)		2A, 2B, 2D, 1A	0.00022–0.00061	8.6–10%
STI (GP)		5B, 4D, 2B, 1A, 6A	0.0001–0.00093	7.7–10.4%
STI (RL)		1B, 6A, 4A, 5B, 5A, UN	0.00034–0.00041	8.1–12.1%
STI (SL)		3A	0.00035–0.00098	8.2–9.3%

In the case of GP, three QTL clusters were observed on 3A (four), 5B (five), and 6B (three). Four major QTL were detected under control conditions with R^2^ ranging from 10.14 (2A) to 19.26% (5B). Under salt stress, two QTL clusters were found on 2B (three) and 5A (two). No major QTLs were reported. The range of allele effects extended from 3.76 to 9.09 under control, while it extended from 3.49 to 4.64% under salt stress (Table S2).

For G%, approximate numbers of SNP markers were found to be significantly associated under control (11) and salt stress (10). Out of the 11 QTL, nine were considered major QTLs (R^2^ ˃10%) (Supplementary Table S3). Remarkably, wsnp_CAP11_rep_c8663_3738968 had an R^2^ of 21.65% (4B) which was the largest major QTL detected in this study with a *p* value of 0.00000002. The allele effects associated with increased G% ranged from 2.2 to 6.82%. Under salt stress, only one major QTL was detected on 2A with an R^2^ of 10.09%. The allele effects associated with increased G% under salt stress extended from 5.0 to 7.96% which was greater than allele effects under control conditions (Table 3). Two QTL clusters were found on 1A (four) and 2A (three) (Table S2).

The largest number of QTLs that were detected under salt stress was accounted for NoR with 17 significant SNPs, while only two SNPs were found to be significantly associated with NoR under control. Two QTL clusters were found on 3A (five) and 7A (two). Four major QTL under salt stress with R^2^ ranged from 11 to 11.97% (Table 3). The two markers detected under control had approximate allele effects, while effects of the alleles associated with NoR ranged from 0.37 to 0.55 under salt stress (Table S2).

Nine and three significant SNPs were found to be associated with RL under control and salt stress, respectively. Two QTLs clusters under control conditions were found 3A (Two) and 7B (two). Three and two major QTLs were found under control (R^2^ = 10.43–11.61%) and salt stress (R^2^ = 10.30–17.56%), respectively (Table 3). The alleles associated with increased RL had effects extending from 1.39 to 2.34 cm and from 1.17 to 1.33 cm under control and salt stress, respectively (Table S2). All QTLs detected under salt stress were minor (R2 < 10%).

For RSR, the number of QTLs under salt stress (four) was higher than those detected under control (two). All the four QTLs were found to be clustered on chromosome 5B. One major QTL was detected under control with an R^2^ of 10.79%.

In SL, two and three QTLs were found under control and salt stress conditions, respectively. All QTLs associated with SL under both conditions were minor (R2 ˂10%) (Table 3). Two QTLs clusters were found under both conditions with two QTLs in each cluster (Table S2).

For the selection indices, significant markers were found to be significantly associated with four indices: SL_STI, G%_STI, GP_STI, and RL_STI. The highest number of QTLs was found for RL_STI with four major QTLs (R^2^ = 10.19–12.12%) (Table 3). Two major QTL were found for STI_G% and STI_GP. Many QTL clusters were observed for the four indices. Three QTL clusters were found for G%_STI (1A, 2A, and 2B) and GP_STI (1A and 5B). Two QTL clusters were found on 1B and 5A for RL_STI. A wide range of allele effects was observed for all four indices (Table S2).

### Common SNP markers

**Fig. 6:**
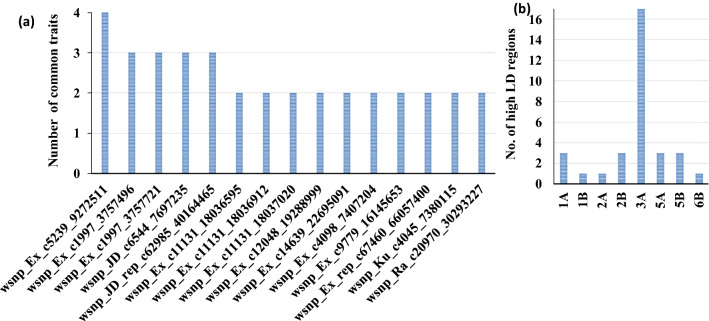
**a** List of common markers which were significantly associated with more than one trait, **b** number of significant LD genomic regions associated with QTLs on each chromosome

Curiously, the common markers which were associated with one more trait are presented in Fig. 6a and Supplementary Table 3. A set of 15 markers were found to be associated with at least two traits. The SNP marker wsnp_Ex_c5239_9272511 was found to be significant in four traits (GP_S, G%_S, GP_STI, and G%_STI), while 10 markers were found to be significantly associated with two traits. These markers were distributed on 1A (three), 1B (one), 2A (three), 1B (one), 3A (one), 5B (six), and 6A (one). In line with the distribution of the significant SNPs on the three genomes A, B, and D, as well as the distribution of QTLs on each chromosome; the 15 common "effective" markers were distributed on the A genome (7) and B genome (8) with 5B chromosome harbors the highest number with 6 markers representing 40% of the effective alleles. No effective alleles were mapped on the D genome.

Notably, two markers wsnp_Ex_c12048_19288999 and wsnp_Ra_c20970_30293227 located on the 2B chromosome were found to be associated with FW under both conditions. Three markers wsnp_Ex_c1997_3757496 (1A), wsnp_Ex_c1997_3757721 (1A), and wsnp_JD_c6544_7697235 (1A) and wsnp_Ex_c5239_9272511 (2B) were found to be associated with G%_STI and GP_STI.

### Linkage disequilibrium (LD)

The markers located on the same chromosome were sorted based on their positions and then the LD (*r*^*2*^*)* among SNPs was calculated. As a result, high LD genomic regions were observed on chromosomes that had significant markers. The LD region locations on the different chromosomes are presented in Fig. 6b. The number of the high significant LD regions ranged from 17 (3A) to one (1B, 2A, and 6B). It was observed that there were some high significant LD regions separated by very low LD markers. For example, on chromosome 2B, five markers were associated with FW_C, the LD region of these five SNPs has been subdivided by wsnp_Ra_c16333_24961476 into two regions although this marker was mapped in the same position of three other markers. Also, some LD regions had all SNPs in a high LD region. For instance, a high significant LD was found among all SNPs associated with G% under salt stress on chromosome 5B.

For the common markers, a complete significant LD was found among the three SNP markers located on 1A. This high LD region was associated with G%_S and G%_STI. Similarly, the three SNPs located on the 2A chromosome were in high significant LD which was associated with G% and G%_STI. On chromosome 5B, two high significant LD regions were found. The first was found between wsnp_Ex_c12048_19288999 and wsnp_Ra_c20970_30293227. This LD region was associated with FW under control and salt stress. The second was found between wsnp_Ex_c11131_18036595 and wsnp_Ex_c11131_18037020 and it was associated with GP_C and GP_STI.

In our study, we focused on the high LD genomic regions harboring markers associated with more than one trait. The gene annotation analysis was performed for the high LD common markers to identify candidate genes residing in this genomic region.

### Gene annotation and expression analysis

The gene annotation was performed on the effective markers (Supplementary Table 3). All the effective SNPs were found to be located within gene models (Fig. 5) that encode important proteins. Noteworthy, the wsnp_Ex_c5239_9272511 SNP marker associated with four traits (G%_S, GP_S, G%_STI, GP_STI), and fell within *TraesCS2B02G135900* that encodes to potassium transporter.

Some high LD regions were found to have the same gene model. For example, all the three SNPs located on chromosome 1A were found to be within the *TraesCS1A02G323600* gene model which encoded to P-loop containing nucleoside triphosphate hydrolase. On chromosome 2A, the high LD genomic region which had SNPs wsnp_Ex_c4098_7407204 and wsnp_Ku_c4045_7380115 was found to be within the *TraesCS2A02G550400* gene model which encoded to zinc finger, CCHC-type superfamily. The other SNP located on the same chromosome had a moderate LD with the aforementioned two SNPs (*r*^*2*^ = 0.51). This SNP fell within a different gene model (*TraesCS2A02G575300*) which encoded a different protein (F-box-like domain superfamily). Moreover, two high LD genomic regions were observed on chromosome 5B. The first region including two SNPs fell within the *TraesCS5B02G400000* gene model that encoded glycosyltransferase family. The second LD region harboring three SNPs wsnp_Ex_c11131_18036595, wsnp_Ex_c11131_18036912, and wsnp_Ex_c11131_18037020 that were located within the *TraesCS5B02G424500* gene model which encoded WPP domain-associated protein.

**Fig. 7:**
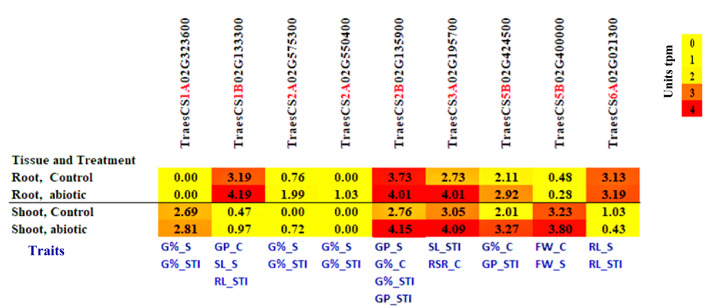
Gene expression of the candidate genes in the roots and shoots under control and abiotic stresses. Units tpm = Transcript per Million

The gene models revealed very different tissue-specific and treatment-specific expression patterns (Fig. 7). *TraesCS1A02G323600* (exclusively expressed in shoot) and *TraesCS2A02G550400* (exclusively expressed in root) exhibiting tissue-specific expressions. The expression of some genes has been exclusively induced under the abiotic stress; *TraesCS2A02G550400* in root and *TraesCS2A02G575300* in shoot exhibiting abiotic-specific expressions. In both root and shoot, the abiotic stress upregulated the gene expression of all gene models except *TraesCS5B02G400000* (in root) and *TraesCS6A02G021300* (in the shoot) that were downregulated. *TraesCS1B02G133300* had the highest expression under abiotic stress in the root, and the *TraesCS2B02G135900* gene had the highest expression level under salt stress.

We compared the gene expression for the identified gene's models in tissue (roots and shoots) under the two treatments control and abiotic stresses. In roots under both conditions, all genes had a higher expression under abiotic stress than under control except *TraesCS1A02G323600* which did not show any expression (tmp = 0), showing moderate shoot-specific expression. *TraesCS2B02G135900* and *TraesCS3A02G195700* genes had the same expression level under abiotic stresses which were significantly different from control for both genes. However, they are associated with different traits; *TraesCS2B02G135900* associated with GP-S, GP_STI, G%_C and G%_STI, while *TraesCS3A02G195700* associated with SL_STI and RSR_C. In shoots under both conditions, all genes had expressed except *TraesCS2A02G550400*. Seven gene models had a higher expression under salt stress than control conditions.

## Discussion

### Genetic variation in salt tolerance at germination and seedling growth stages

Germination is a sensitive growth stage to salt stress and is considered an important growth stage in the plant’s life (Feizi and Aghakhani 2007; Moursi 2014; Wu et al. 2019). Therefore, evaluating genotypes at this stage is very effective in abiotic stress studies to develop tolerant cultivars (Sallam et al. 2019). In our study, a set of 176 genotypes covering 22 countries was tested for salt tolerance at seed germination and early growth stage. The high genetic variation among genotypes revealed by ANOVA is very useful for wheat breeders and geneticists for further improvement of salt tolerance. The significant differences between the two treatments indicated that exposing genotypes to NaCl affected plant performances compared to control. The significant G × T interactions in all traits indicated genotypes respond differently under both treatments. The high heritability estimates for all traits promised with fruitful selection for salt tolerance in the current plant material. Screening this population for tolerance to various abiotic stresses is one of the main objectives of breeding programs in Egypt. In another study, using different assessment indices, the same set of genotypes presented high genetic variation in drought tolerance at the seedling stage (Ahmed et al. 2021). Therefore, the plant material of this study is expected to have a large genetic variation in abiotic stresses tolerance which will be useful for further genetic studies.

Selection indices presented very clear discrimination between tolerant and susceptible genotypes (Fig. 1). Five selection indices, representing germination and seedling traits, were calculated for G%, GP, FW, SL, and RL. The most salt-tolerant genotypes differed by trait. Stress tolerance indices of the same traits have been successfully used to discriminate for salt tolerance in wheat and spring barley (Oyiga et al. 2016; Sallam et al. 2019; Thabet et al. 2021).

### Phenotypic correlation

The phenotypic correlations among G% and GP under both conditions were always negative especially under salt stress, suggesting different genetic control. In contrast, the correlations among SL, RL, RSR, and FW were positive except between SL and RSR under both treatments. In barley, at the same stages, the germination-related traits showed negative correlations while the seedling development traits were positively correlated under salt and drought stresses (Thabet et al. 2018, 2021; Moursi et al. 2020a). The correlations of the germination-related traits (G%, GP) with the seedling-related traits (SL, RL, RSR, FW) were non-significant or negative. These findings indicate that it may be possible that seed germination and seedling establishment are under independent genetic control in wheat.

### Most promising drought-tolerant genotypes

As discussed in the previous part, the weak or no significant correlation among the stress tolerance indices was an obstacle to finding the most salt-tolerant genotypes in all indices. First, all genotypes were sorted from the most tolerant to susceptible in each index. Second, the top 10 most tolerant genotypes were selected from each index. No tolerant genotype based on GP_STI was characterized as tolerant in the other indices based on the second criteria. Therefore, GP_STI was excluded due to the lack of correlation with SL_STI, RL_STI, and FW_STI on one hand, and a negative significant correlation with G%_STI. The remaining four indices were divided into two groups, germination (G%_STI) and seedling (RL_STI, SL_STI, and FW_STI) indices. For the tolerant genotypes, all genotypes were salt tolerant based on indices estimated from germination and seedling traits, especially PI 201414 (Australia) that was among the most salt tolerant genotypes for SL_STI and FW_STI. Crossing among these genotypes could be useful for producing wheat cultivars with high salt tolerance at germination and seedling stages. PI 525241 from Morocco was the best promising genotype that could be included in all crosses as a salt tolerant candidate parent as it was among the most salt tolerant genotypes in three indices estimated from germination (G%) and seedling (RL and FW) traits (Table 2). Moreover, PI 525241 was the only genotype among the tolerant group for RL_STI. It was reported that salt tolerant genotypes tend to have longer roots than susceptible genotypes (An et al. 2003). Bearing in mind that the tolerant genotypes were from different countries, therefore, improving salt tolerance can be achieved on one hand, and the level of genetic diversity will be increased on the other hand. It is highly recommended to use more than stress tolerance trait to have an accurate phenotypic selection at the target growth stage, especially for those traits that have weak or no correlations (Sallam et al. 2015, 2018).

The most susceptible salt tolerant genotypes were also studied. These susceptible genotypes can be used as checks for further salt tolerance experiments or as the susceptible parent in crosses seeking the development of double haploid populations. Remarkably, Sohag-5 was characterized as salt susceptible. The same genotype was previously reported as a salt susceptible genotype at the germination stage by Gowayed and Abd El-Moneim (2021).

Genotypes with salt tolerance features may not be tolerant at adult growth stage. Therefore, it is highly recommended to evaluate all genotypes used in this study under field conditions with salty soils to study the reduction in agronomic traits. For this purpose, this collection is being currently tested under field conditions in saline soil in Egypt. Using controlled environments, line selection can be successfully performed through consistency of the environment (Ali and Johnson 2000). Therefore, selecting the best genotypes for salt tolerance could be based on their performances in the growth chamber and then following with selection in the field (Dawood et al. 2020).

### Genome-wide association study for salt tolerance

Salt tolerance is a complex trait that is controlled by many genes (Oyiga et al. 2018; Thabet et al. 2021). The GWAS is a powerful analysis that can be used to dissect polygenic traits (Alqudah et al. 2020). This method was used to identify markers associated with salt tolerance at early growth stages in some of the important cereals such as barley (Mwando et al. 2020; Hu et al. 2021; Thabet et al. 2021) and rice (Yu et al. 2018). Unfortunately, there were very few studies on GWAS for identifying genetic alleles associated with salt tolerance under early growth stages in spring Chinese and winter wheat, reported by Yu et al. (2020) and Oyiga et al. (2018), respectively. Therefore, more studies and efforts should be made to identify genes controlling salt tolerance at germination and seedling stages. Here, we used diverse spring wheat genotypes collected from 22 different countries representing five continents (Africa, Europe Asia, Australia/Oceania, North America) (supplementary Table 1). This diverse collection offers an opportunity of identifying new QTL controlling salt tolerance in spring wheat. The analysis of PCA revealed the distribution of these genotypes based on their genetic distance (supplementary Table 2). Eight genotypes from Asia have separated away from all genotypes. A set of 103 genotypes from the current population was extensively analyzed for genetic diversity and population structure by Mourad et al. (2020) using 36,720 SNP markers. The population structure divided the genotypes into three possible subpopulations (Mourad et al. 2020). The PCA analysis performed by Mourad et al. (2020) was approximately similar with the PCA results of this study using the 6141 SNP markers. Additionally, (Ahmed et al. 2021) analyzed PCA based on the genetic distance among a set of 138 genotypes from the set used in this study using 407 DArT markers. The PCA results from Ahmed et al. (2021) separated seven genotypes away from all other genotypes. Therefore, the PCA in this study was included in the GWAS model to avoid any spurious association due to the population structure.

In this study, GWAS was successful for detecting 138 significant markers associated with germination and seedling traits under both treatments with more QTLs under salt stress (49) than normal (42) at *p* < 0.001. At 0.2 FDR, SNPs were detected for only four traits (Fig. 4a). The QQ plot indicated that the GLM + PCA model effectively controls the false positive. The 0.2 FDR as a significant level for marker-trait association was used before to identify 25 significant SNPs associated with frost tolerance in highly diverse winter faba bean populations by Sallam et al (2016) who succeeded to validate five SNPs in a different genetic background (101 recombinant inbred lines).

A set of 130 QTL was detected for salt tolerance at germination and seedling stages under salt and normal conditions (Yu et al. 2020). The highest number of significant SNPs were found to be associated with NoR at p > 0.001 and 0.2 FDR with 17 and FDR 10 markers under salt stress, respectively. Therefore, NoR was considered the most important trait in this study under salt stress.

Most of QTL were found to be located on chromosome 5B in this study. Moreover, genome A and B possessed an equal number of QTLs detected in this study with 64 in each. A similar finding was reported by Oyiga et al. (2018) who found the highest number of QTL on chromosome 5B and a nearly equal number of QTLs in genomes A (72) and B (67). This indicated that genome B of *Aegilops* section Sitopsis may be a good source of salt tolerance in wheat. The Ph inhibitor line derived from *Ae. speltoides* (Chen and Tsujimoto 1994) was the source of tissue tolerance to salinity in salt-tolerant spring wheat lines W4909 and W4910 (Wang et al. 2003; Mott and Wang 2007; Genc et al. 2019). In the study of Yu et al., (2020), most SNPs (N = 117) associated with salt tolerance index for germination rate were located on 1A. The physical positions of the SNPs detected for G% in this study were compared with those detected by Yu et al. 2020, and the results indicated that all SNPs in the two studies are in different physical positions (long genetic distance). This indicated that the QTLs detected for G% in this study were novel and added more information on genes and markers associated with germination rate under salt stress. It was difficult to compare the position of QTL detected in this study and Oyiga et al. (2018) as the QTLs in the latter study were mapped in cM and no physical position was available. However, both studies shared the same SNPs.

Out of 138 significant SNPs, 30 were found to have R^2^ > 10% which indicated that these QTLs were with major effects. Both significant levels allowed detecting markers with minor and large effects. Zhao et al. (2018) reported QTL with R^2^ ranged from 7.27 to 13.31% using p > 0.001 and 10% FDR in a rice GWAS panel tested under AL stress. At 20% FDR, minor effects of QTL associated with frost tolerance (R^2^ < 10%) were detected and validated in two different genetic backgrounds of faba bean (Sallam et al. 2016). It is very important to identify QTLs with minor and major effects with different significant thresholds to dissect the genetics of complex traits such as salt tolerance.

The LD among significant SNPs provide very useful information. A set of 46 high LD genomic regions were found indicating that these high LD SNPs tend to be co-inherited together from generation to generation, while the low LD regions represented individual QTLs. The test of LD is very important as it will save time and effort in selecting the SNP markers that can be used for marker-assisted selection and further markers validation studies (Mourad et al. 2018; Eltaher et al. 2021).

Remarkably, SNP markers (n = 15) associated with more than one trait were detected in this study. These 15 markers have pleiotropic effects at one locus. This result supports the prospects of marker-assisted selection choosing highly promising markers for improving salt tolerance in spring wheat. The common markers were located on the same chromosome which indicated that these genomic regions represented one QTL. Expectedly, as there was a very weak or no significant correlation between germination and seedling stage, no significant SNPs were found to be associated with the two types. However, one SNP marker wsnp_JD_rep_c62985_40164465 located on 1B chromosome was found to be associated with GP, SL, and RL_STI. To avoid false positive association due to the relaxed FDR significant level, these markers can be converted to KASP markers to be validated in different wheat populations. All SNPs detected in this study should be validated in a different genetic background before being used in marker-assisted selection in wheat breeding programs to improve salt tolerance (Mourad et al. 2019; Moursi et al. 2020a, b). SNP markers with pleiotropic effects on salt tolerance across growth stages were reported by Oyiga et al. (2018).

### Gene annotation and gene expression

The gene annotation has been conducted only for the effective SNP markers (SNPs with pleiotropic effect i.e. SNPs controlling more than one trait). The A and B genomes harbored the effective markers; especially chromosome 5B that harbored 5 effective SNPs out of 15 SNPs. Similarly, several pleiotropic loci were detected in wheat under salinity stress and the B genome harbored the highest number of markers associated with salinity tolerance and other agronomic traits, especially chromosome 5B (Amin and Diab 2013; Rahimi et al. 2019; Hu et al. 2021). No effective SNPs were identified in the D genome; which might be attributed to the low polymorphism that has been found in the D genome (Röder et al. 1998; Semagn et al. 2006).

The gene annotation showed that the candidate genes belong to different functional proteins including but not limited to potassium transporter and Zinc finger, CCHC-type superfamily. All genes have been upregulated under the abiotic stress except *TraesCS5B02G400000* (in root) and *TraesCS6A02G021300* (in the shoot). All genes exhibited constitutive expression patterns (expressed under control and abiotic stress) except *TraesCS2A02G550400* on chromosome 2A that showed an adaptive expression pattern.

In the present study, the gene *TraesCS1A02G323600* showed an association with G%_S and G%_STI (Fig. 7; Table S3). This gene encoding P-loop containing nucleoside triphosphate hydrolase showed a tissue-specific expression as being exclusively expressed in the shoot (Fig. 7). In wheat, the P-loop motifs were found to be important components of the heat shock protein that confers heat tolerance (Erdayani et al. 2020). In Arabidopsis, the P-loop protein was highly expressed in shoot meristems and was involved in the enhancement of cell division via regulating the reactive oxygen species (ROS) homeostasis (Yu et al. 2016b). The P-loop played a key role in salinity tolerance in various species including Arabidopsis, Alfalfa, and rice (Cheung et al. 2013; Liu et al. 2019). This gene exhibited a constitutive expression pattern (expressed under control and abiotic stress), agreeing with the finding that P-loop coding genes showed a constitutive expression under salinity in rice (Cotsaftis et al. 2011). The upregulation of this gene under salt stress suggests that this gene is involved in salinity tolerance via promoting G% by increasing the cell division rate. The gene *TraesCS1B02G133300* (associated with SL and RL_STI) encoded phosphodiesterase, TIM beta/alpha-barrel domain superfamily has been upregulated in root and shoot under salinity stress (Fig. 7). Congruent with our findings, the genes encoding this protein family has been expressed in all organs of Arabidopsis and wheat under salinity and drought (Hirayama et al. 1995; Zhang et al. 2014). Moreover, inhibiting the expression of these genes caused low seedling growth and low resistance to salinity and drought (Zhang et al. 2014). On chromosome 2A, the gene *TraesCS2A02G550400* (associated with G%_S and G%_STI) showed a unique pattern; it has been exclusively induced under abiotic stress in the root. This gene encodes a Zinc finger, a CCHC-type superfamily. The wheat Zinc finger families played various roles in seed germination and seedling development under salinity and drought stress in Arabidopsis (Xu et al. 2014). Another gene on chromosome 2A, *TraesCS2A02G575300* (associated with G%_S and G%_STI and encoded F-box-like domain superfamily) has been overexpressed in root and shoot under abiotic stress. The overexpression of wheat F-box improved salinity tolerance in tobacco through improving germination rate and root elongation (Zhao et al. 2017). Notably, on the 2B chromosome, the gene *TraesCS2B02G135900* associated with the highest number of traits (Fig. 7, Table S3) and encoded a Potassium (K^+^) transporter. In rice, the overexpression of OsHAK21 (a K^+^ transporter) under salinity stress enhanced seed germination and seedling establishment (He et al. 2019). Two QTLs with positive additive effects for K^+^ uptake were mapped on the 2B chromosome during the seedling stage under salinity stress (Xu et al. 2013), Similarly, one QTL with a positive additive effect was mapped on the 2B chromosome in a doubled haploid wheat population (Excalibur × Kukri) (Asif et al. 2018). These findings suggest that the 2B chromosome harbors potential genes and alleles for K^+^ uptake in wheat.

On chromosome 5B, the gene *TraesCS5B02G424500* (associated with GP_C and GP_STI, and coded WPP domain-associated protein). As this gene is associated with GP, probably, this gene encodes proteins that are involved in cell division. The WPP is necessary for the plant cell mitotic division (Jeong et al. 2005). In Arabidopsis, the WPP1 and WPP2 were found to be associated with the nuclear envelope and cell plate formations particularly in the undifferentiated cells of the root tip. Suppression of the Arabidopsis WPP family impaired the mitotic division resulting in shorter primary roots (Patel et al. 2004). In agreement with our findings, the upregulation of WPP under salt stress has been reported in root cortical cells of Arabidopsis and rice (EVRARD 2012), in pepper (Park et al. 2017). The second gene on chromosome 5B, *TraesCS5B02G400000* associated with FW_C and FW_S, and encoded Glycosyl transferase, family 35. Amin and Diab (Amin and Diab 2013) reported that the chromosomes 2B and 5B harbor potential alleles that control the variation of several traits including K^+^/Na^+^ uptake. Unlike the remaining genes, this gene exhibited tissue-specific expression; it has been downregulated in roots and upregulated in shoots under abiotic stress relative to control (Fig. 7). The ecotypic expression of Arabidopsis glycosyltransferase improved salinity tolerance in tobacco by exhibiting a better germination rate and better plant growth (Sun et al. 2013). The glycosyltransferase is involved in the biosynthesis of xylan, the major constituent of the cell wall and represents the largest portion of plant biomass (Rennie et al. 2012). This supports our findings as this gene is associated with the variation of FW under control and salinity stress.

On chromosome 3A, the gene *TraesCS3A02G195700* associated with SL_STI and RSR_C, and encoded LNK family. This gene had the highest increase in gene expression under abiotic stress relative to control in both shoots and roots (Fig. 7). The genes of this family belong to the circadian clock genes that fine-tune the plant response to biotic and abiotic stimuli including heat and salinity (Nagel et al. 2015). This protein family is well known as regulators of light regulation of the flowering controlling genes. However, they have been regulated under abiotic stresses in oil palm (reviewed in (Ooi et al. 2021)). In Arabidopsis, six genes belonging to the LNK family were found to be involved in stem elongation (Rugnone et al. 2013).

On chromosome 6A, the gene *TraesCS6A02G021300* associated with RL_S and RL_STI, and encoded S-adenosyl-L-methionine-dependent methyltransferase. This gene showed tissue-specific expression patterns; it has been upregulated in root and downregulated in shoot suggesting a potential role in root development under abiotic stress. This is supported by the positive correlation between RL_S and FW_S (Fig. 2). Salt stress enhanced the expression of S-adenosyl-L-methionine synthase and xylem development in the roots of tomato plants (Sánchez-Aguayo et al. 2004). The overexpression of S-adenosyl-L-methionine synthetase (SAM) (a key enzyme in S-adenosyl-L-methionine, a precursor to polyamine and ethylene biosynthesis) in tomato enhanced tolerance to salinity and alkalinity through enhancing well-developed roots (Gong et al. 2014).

Specific primers can be designed for these genes for further experiments to validate the expression of these genes using real-time PCR under control and salt stress at seedling stage.

## Validation of SNP markers associated with salt tolerance

Interestingly, one marker wsnp_Ex_rep_c101323_86702546 (5A) was found to be significant in both studies. This marker was significantly associated with salt tolerance for grain yield in wheat (Oyiga et al. 2018), while it was associated with the increased number of roots (NOR) under salt stress in our study. The allele C of this marker was associated with increases of the two traits under salt stress. This marker may be associated with salt tolerance under seedling and adult growth stages. Therefore, this marker should be included among the most important markers detected in this study as the validation was done in a population with a different genetic background.

This validated SNP was found to be located in *TraesCS5A02G527600* gene model which encoded NADPH oxidase respiratory burst protein. Salt stress activates NADPH oxidases at the transcriptional and functional levels (Ma et al. 2012) and is an efficient, self-amplifying mechanism, forming a so-called “ROS-Ca^2+^ hub” (Demidchik and Shabala 2018). Moreover, it was reported that root respiratory burst oxidase homolog (RBOH)-dependent H_2_O_2_ production played an important role in salt tolerance by controlling stomatal closure and the exclusion of Na^+^  and, thus optimizing plant ionic and water balance under salt stress conditions (Niu et al. 2018). This conclusion further supports our GWAS results as the same marker was associated with the increased number of roots under salt stress.

## Conclusion

Salinity stress significantly reduced all seed germination parameters and seedling development-related traits. The high diverse genotypes that represented five continents and 22 different countries possessed a broad genetic variation. This high genetic variation can be used for breeding to genetically improve salt tolerance in wheat at early growth stages. Very promising salt tolerant and highly diverse genotypes were selected for future breeding programs to improve salt tolerance in spring wheat. The genome-wide association study revealed novel and very important genomic regions that had important gene models controlling salt tolerance. More importantly, the SNP marker wsnp_Ex_rep_c101323_86702546 was validated in our study with its strong association with salt tolerance. This marker resides in a gene model which encoded an important protein that plays an important role in Na^+^ exclusion and alleviating the effect of salt stress. The gene expression data supported the strong association between the SNP markers and salt tolerance and indicated that a gene expression database is a powerful tool for providing useful information on the expression of target genes. The SNP markers detected in this study are very important in future marker-assisted selection for accelerating breeding programs to improve salt tolerance in wheat. Moreover, the results of this study provided useful information for future breeding work and helped in understanding the genetic architecture of salt tolerance at germination and seedling growth stages.

## Supplementary Information

Below is the link to the electronic supplementary material.

Supplementary file1 (PPTX 1087 kb)

Supplementary file2 (XLSX 667 kb)

## Data Availability

All relevant data can be found within the manuscript and its supporting materials.
