# Supplementary material for: High-LD SNP markers exhibiting pleiotropic effects on salt tolerance at germination and seedlings stages in spring wheat
Source: Plant Mol Biol. 2022 Feb 25;108(6):585–603. doi: 10.1007/s11103-022-01248-x (PMC8967789; doi:10.1007/s11103-022-01248-x)
Supplement: Supplementary file 1 — Supplementary file1 (PPTX 1087 kb) [file 11103_2022_1248_MOESM1_ESM.pptx]

## Slide 1
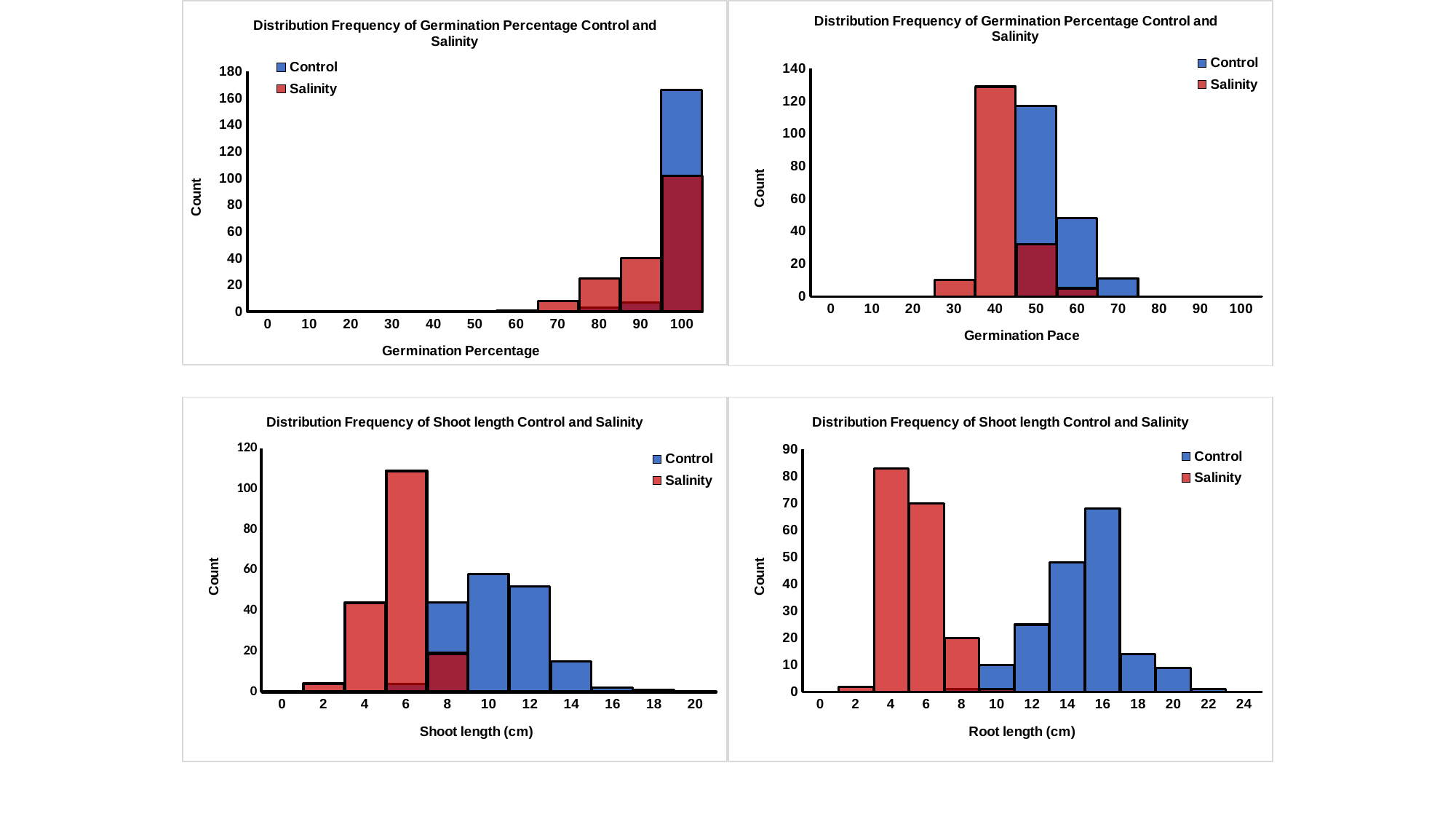

### Chart: Distribution Frequency of Germination Percentage Control and Salinity
| Category | | |
|---|---|---|
| 0 | 0.0 | 0.0 |
| 10 | 0.0 | 0.0 |
| 20 | 0.0 | 0.0 |
| 30 | 0.0 | 0.0 |
| 40 | 0.0 | 0.0 |
| 50 | 0.0 | 0.0 |
| 60 | 0.0 | 1.0 |
| 70 | 0.0 | 8.0 |
| 80 | 3.0 | 25.0 |
| 90 | 7.0 | 40.0 |
| 100 | 166.0 | 102.0 |
### Chart: Distribution Frequency of Germination Percentage Control and Salinity
| Category | | |
|---|---|---|
| 0 | 0.0 | 0.0 |
| 10 | 0.0 | 0.0 |
| 20 | 0.0 | 0.0 |
| 30 | 0.0 | 10.0 |
| 40 | 0.0 | 129.0 |
| 50 | 117.0 | 32.0 |
| 60 | 48.0 | 5.0 |
| 70 | 11.0 | 0.0 |
| 80 | 0.0 | 0.0 |
| 90 | 0.0 | 0.0 |
| 100 | 0.0 | 0.0 |
### Chart: Distribution Frequency of Shoot length Control and Salinity
| Category | | |
|---|---|---|
| 0 | 0.0 | 0.0 |
| 2 | 0.0 | 4.0 |
| 4 | 0.0 | 44.0 |
| 6 | 4.0 | 109.0 |
| 8 | 44.0 | 19.0 |
| 10 | 58.0 | 0.0 |
| 12 | 52.0 | 0.0 |
| 14 | 15.0 | 0.0 |
| 16 | 2.0 | 0.0 |
| 18 | 1.0 | 0.0 |
| 20 | 0.0 | 0.0 |
### Chart: Distribution Frequency of Shoot length Control and Salinity
| Category | Control | Salinity |
|---|---|---|
| 0 | 0.0 | 0.0 |
| 2 | 0.0 | 2.0 |
| 4 | 0.0 | 83.0 |
| 6 | 0.0 | 70.0 |
| 8 | 1.0 | 20.0 |
| 10 | 10.0 | 1.0 |
| 12 | 25.0 | 0.0 |
| 14 | 48.0 | 0.0 |
| 16 | 68.0 | 0.0 |
| 18 | 14.0 | 0.0 |
| 20 | 9.0 | 0.0 |
| 22 | 1.0 | 0.0 |
| 24 | 0.0 | 0.0 |

## Slide 2
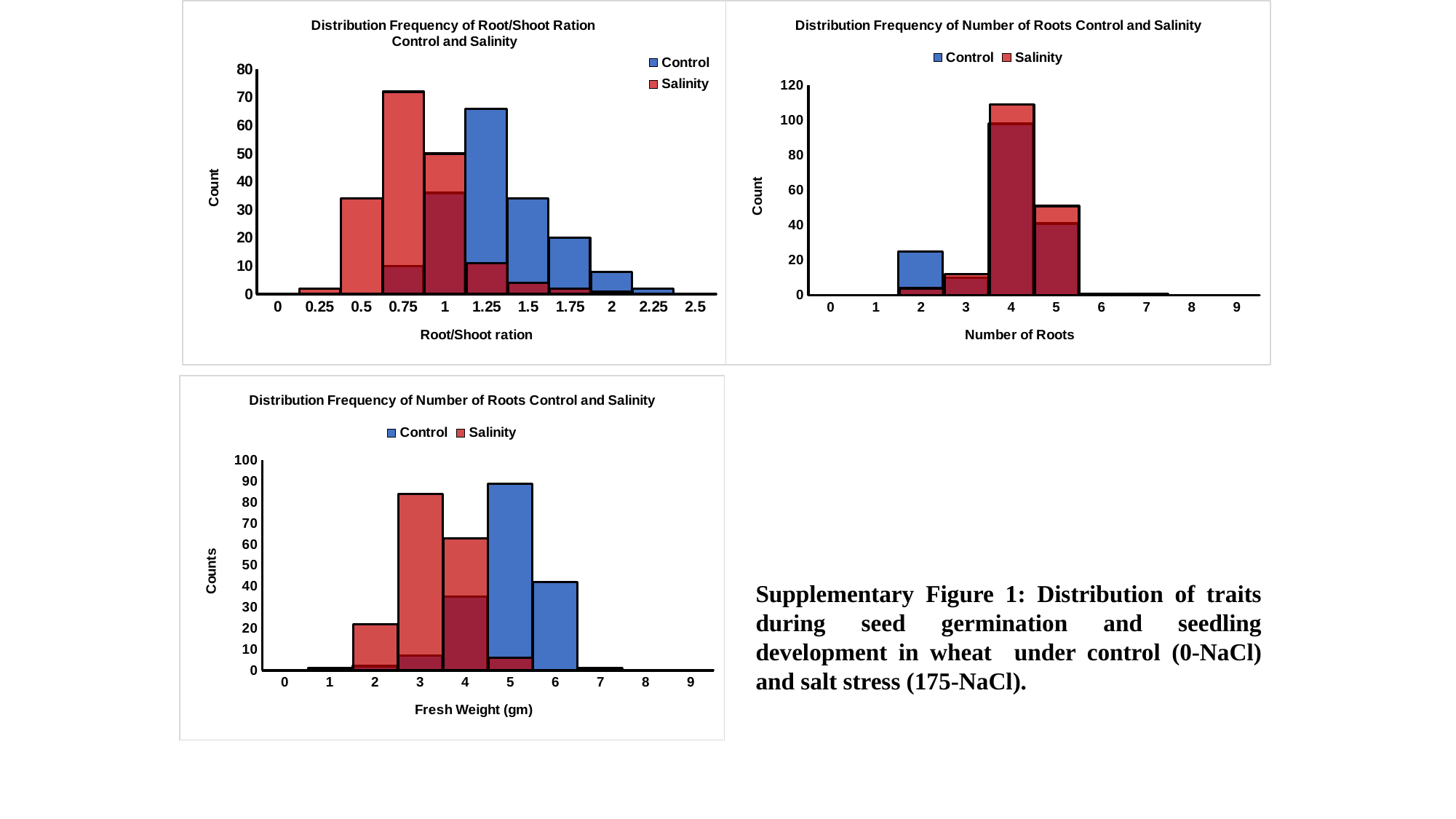

### Chart: Distribution Frequency of Root/Shoot Ration
Control and Salinity
| Category | Control | Salinity |
|---|---|---|
| 0 | 0.0 | 0.0 |
| 0.25 | 0.0 | 2.0 |
| 0.5 | 0.0 | 34.0 |
| 0.75 | 10.0 | 72.0 |
| 1 | 36.0 | 50.0 |
| 1.25 | 66.0 | 11.0 |
| 1.5 | 34.0 | 4.0 |
| 1.75 | 20.0 | 2.0 |
| 2 | 8.0 | 1.0 |
| 2.25 | 2.0 | 0.0 |
| 2.5 | 0.0 | 0.0 |
### Chart: Distribution Frequency of Number of Roots Control and Salinity
| Category | Control | Salinity |
|---|---|---|
| 0 | 0.0 | 0.0 |
| 1 | 0.0 | 0.0 |
| 2 | 25.0 | 4.0 |
| 3 | 10.0 | 12.0 |
| 4 | 98.0 | 109.0 |
| 5 | 41.0 | 51.0 |
| 6 | 1.0 | 0.0 |
| 7 | 1.0 | 0.0 |
| 8 | 0.0 | 0.0 |
| 9 | 0.0 | 0.0 |
### Chart: Distribution Frequency of Number of Roots Control and Salinity
| Category | Control | Salinity |
|---|---|---|
| 0 | 0.0 | 0.0 |
| 1 | 0.0 | 1.0 |
| 2 | 2.0 | 22.0 |
| 3 | 7.0 | 84.0 |
| 4 | 35.0 | 63.0 |
| 5 | 89.0 | 6.0 |
| 6 | 42.0 | 0.0 |
| 7 | 1.0 | 0.0 |
| 8 | 0.0 | 0.0 |
| 9 | 0.0 | 0.0 |Supplementary Figure 1: Distribution of traits during seed germination and seedling development in wheat under control (0-NaCl) and salt stress (175-NaCl).

## Slide 3
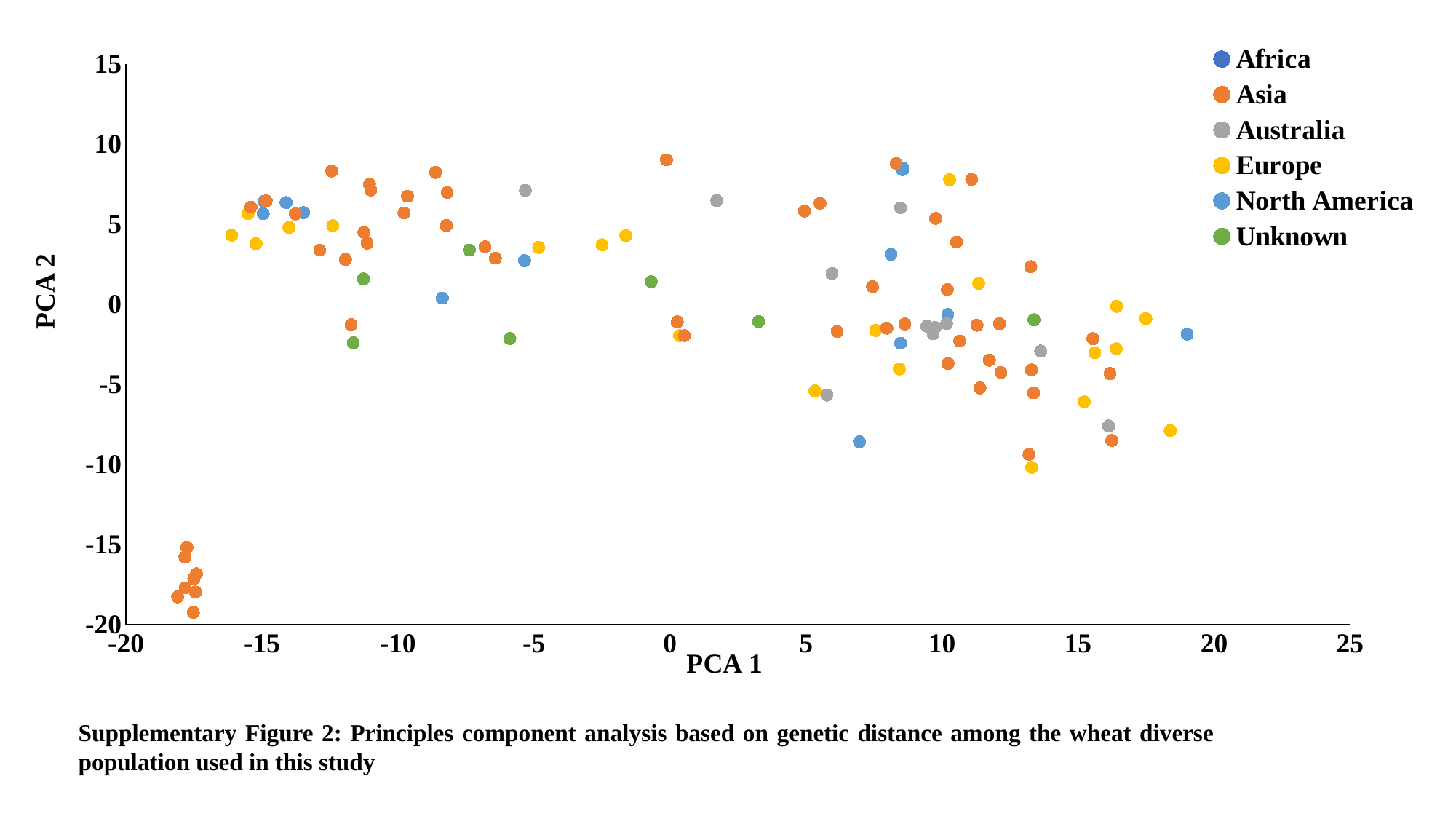

### Chart
| Category | | | | | | |
|---|---|---|---|---|---|---|Supplementary Figure 2: Principles component analysis based on genetic distance among the wheat diverse population used in this study

## Slide 4
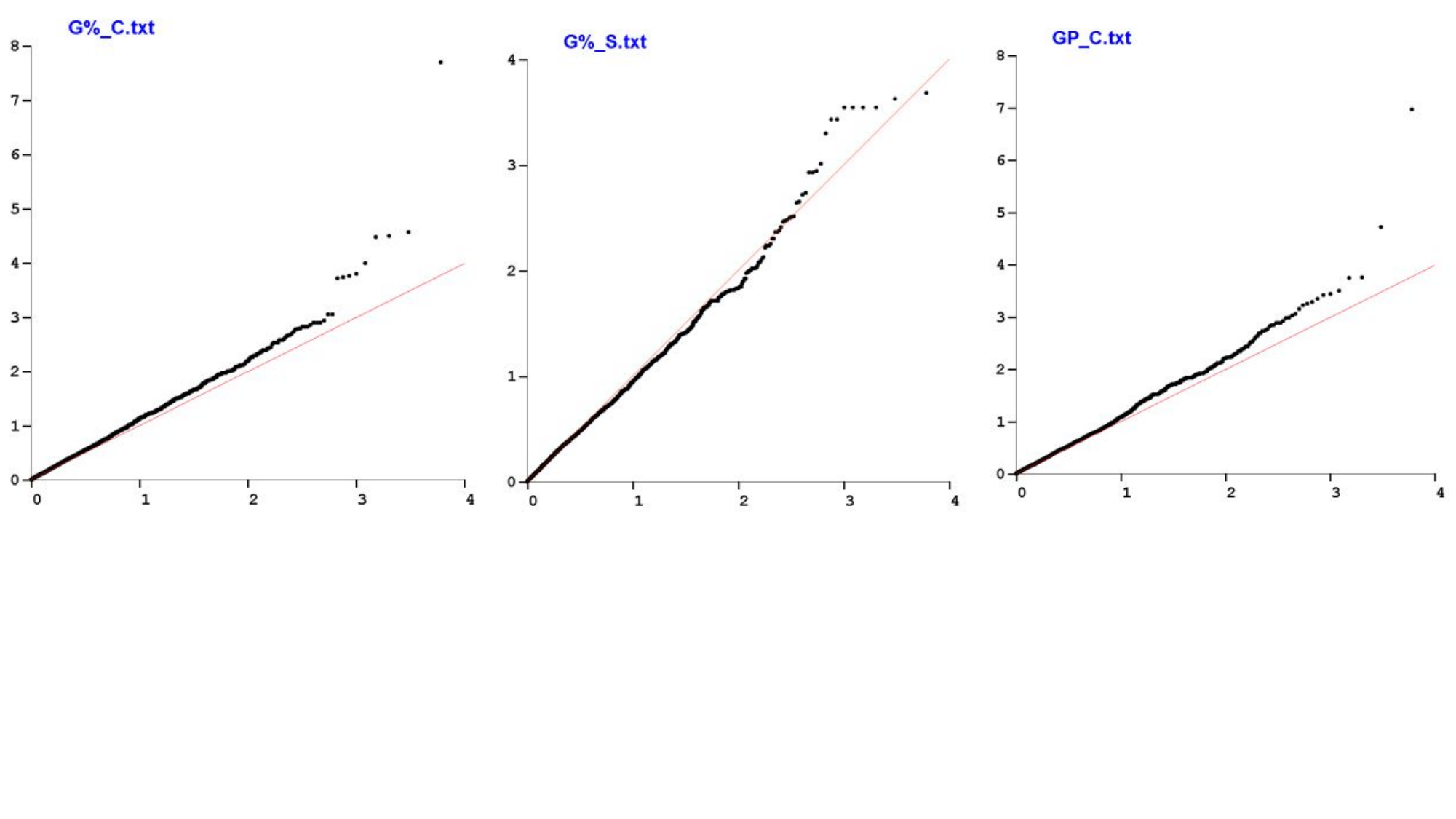

## Slide 5
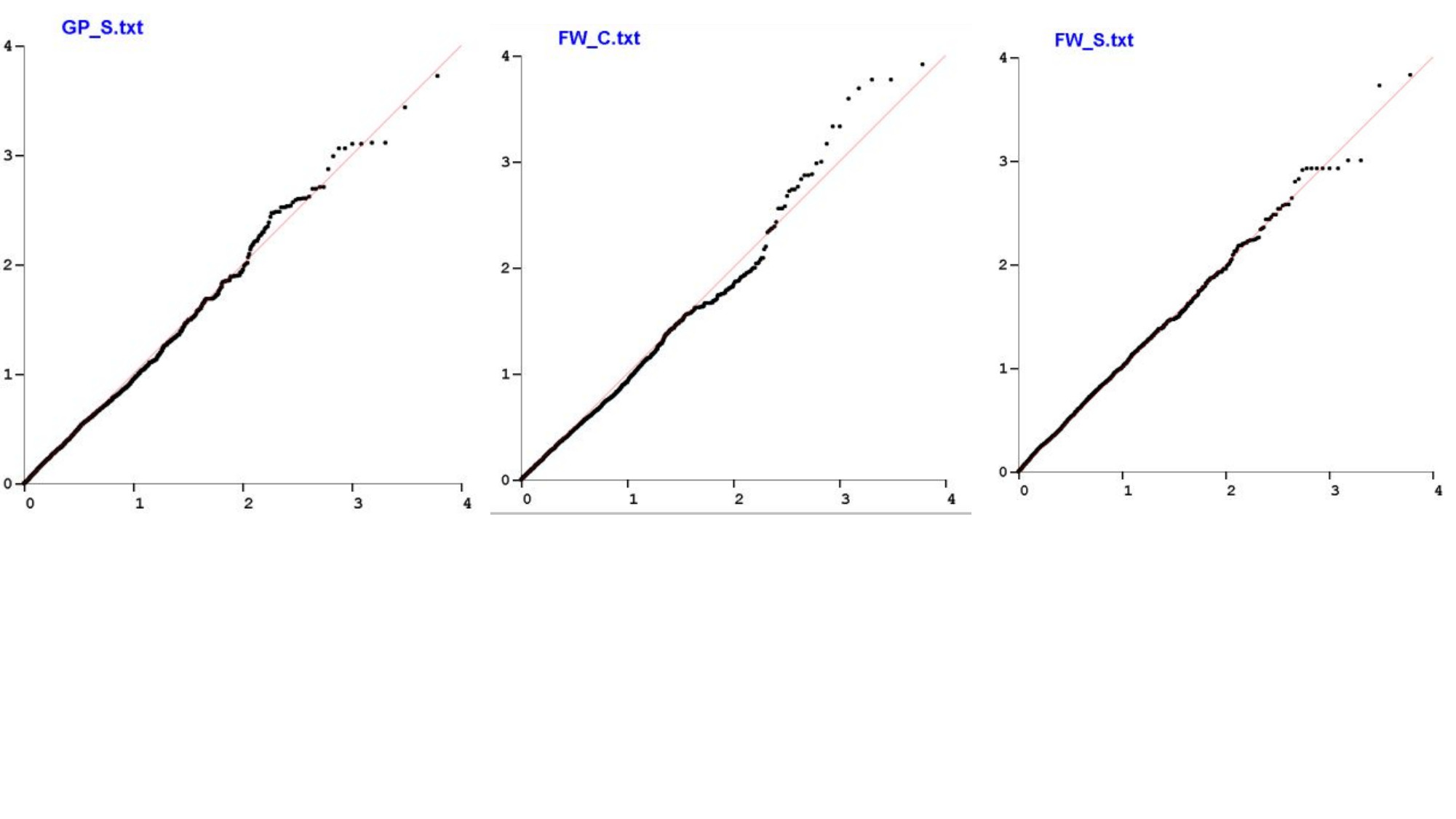

## Slide 6
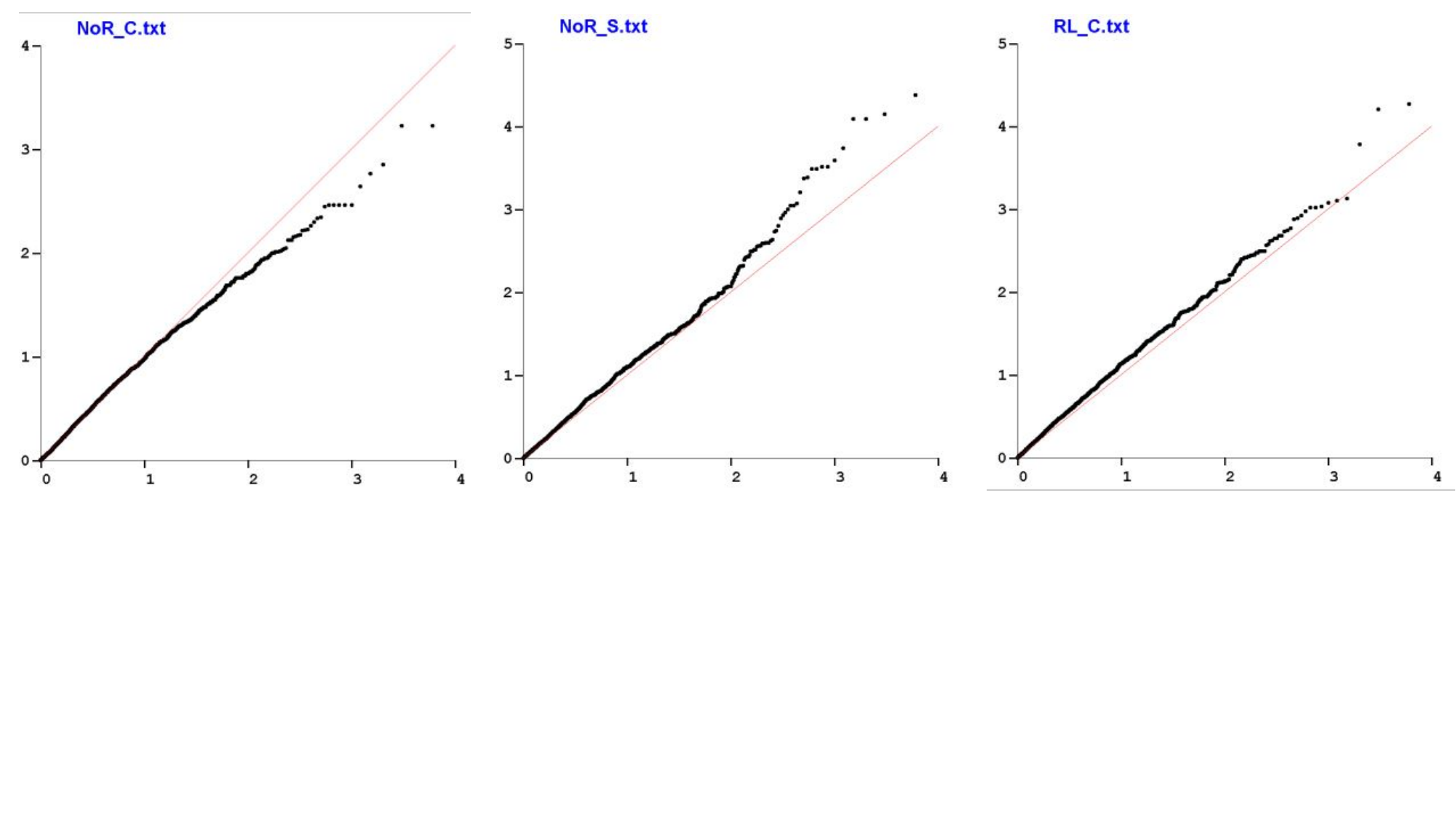

## Slide 7
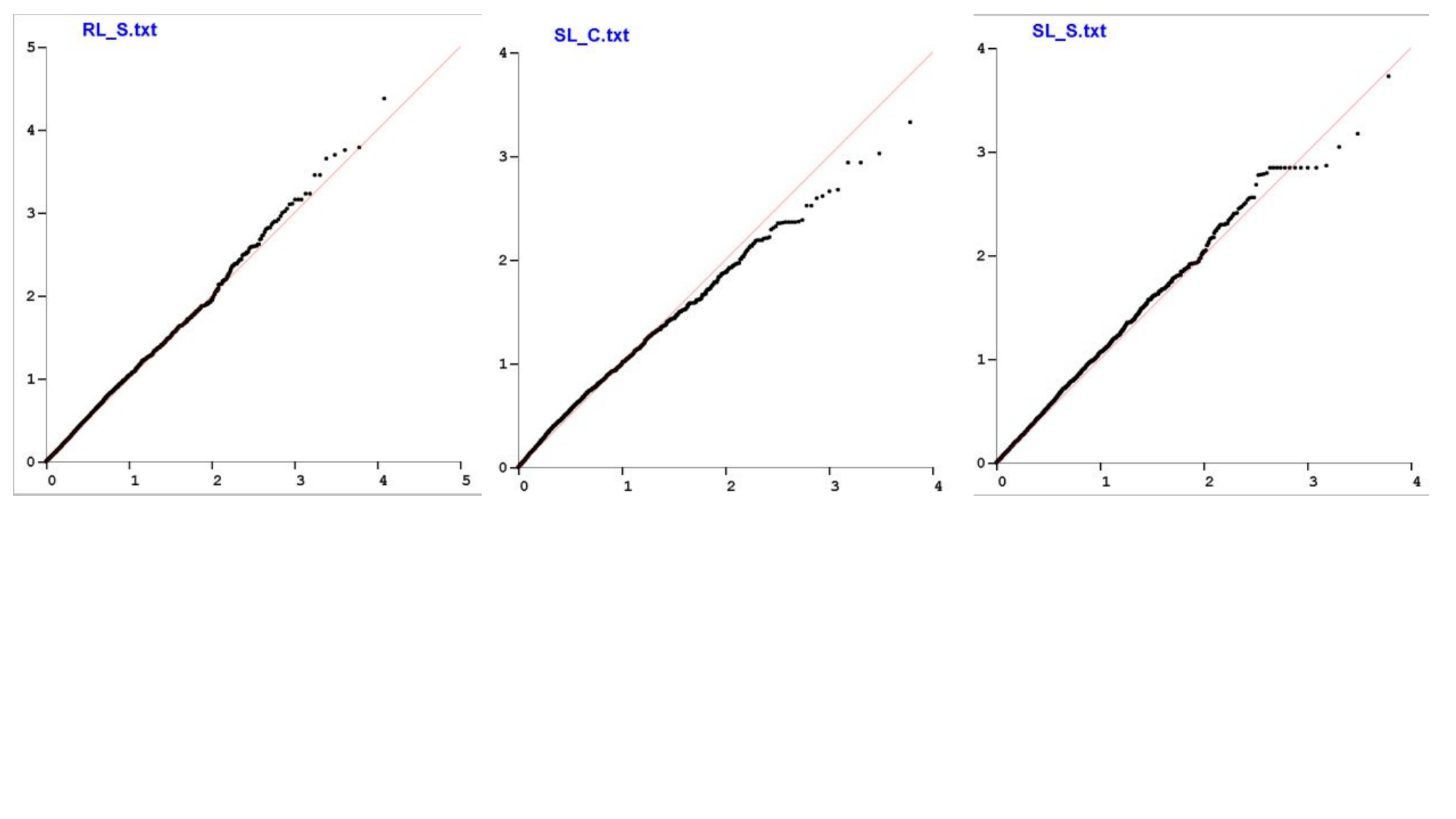

## Slide 8
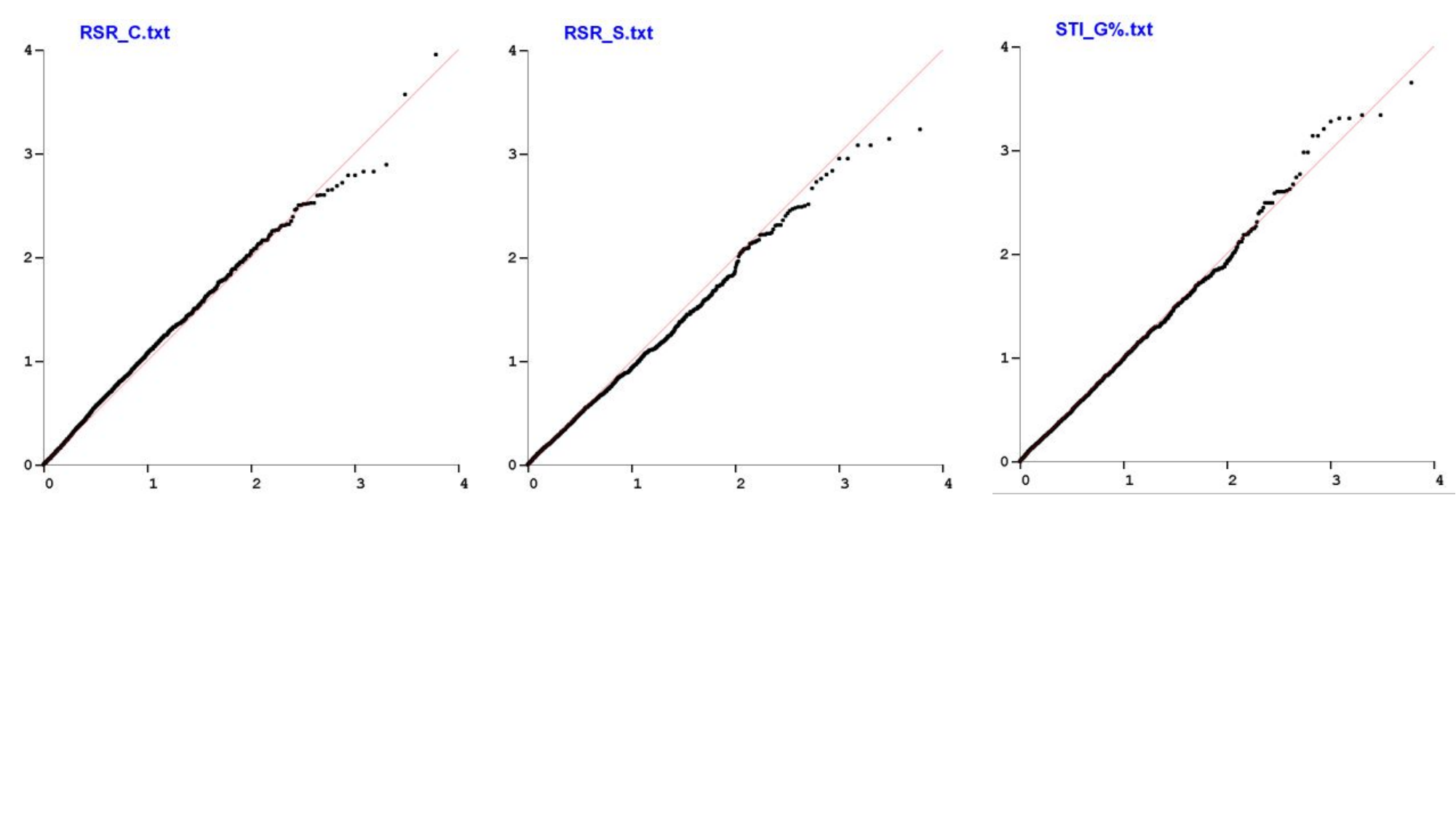

## Slide 9
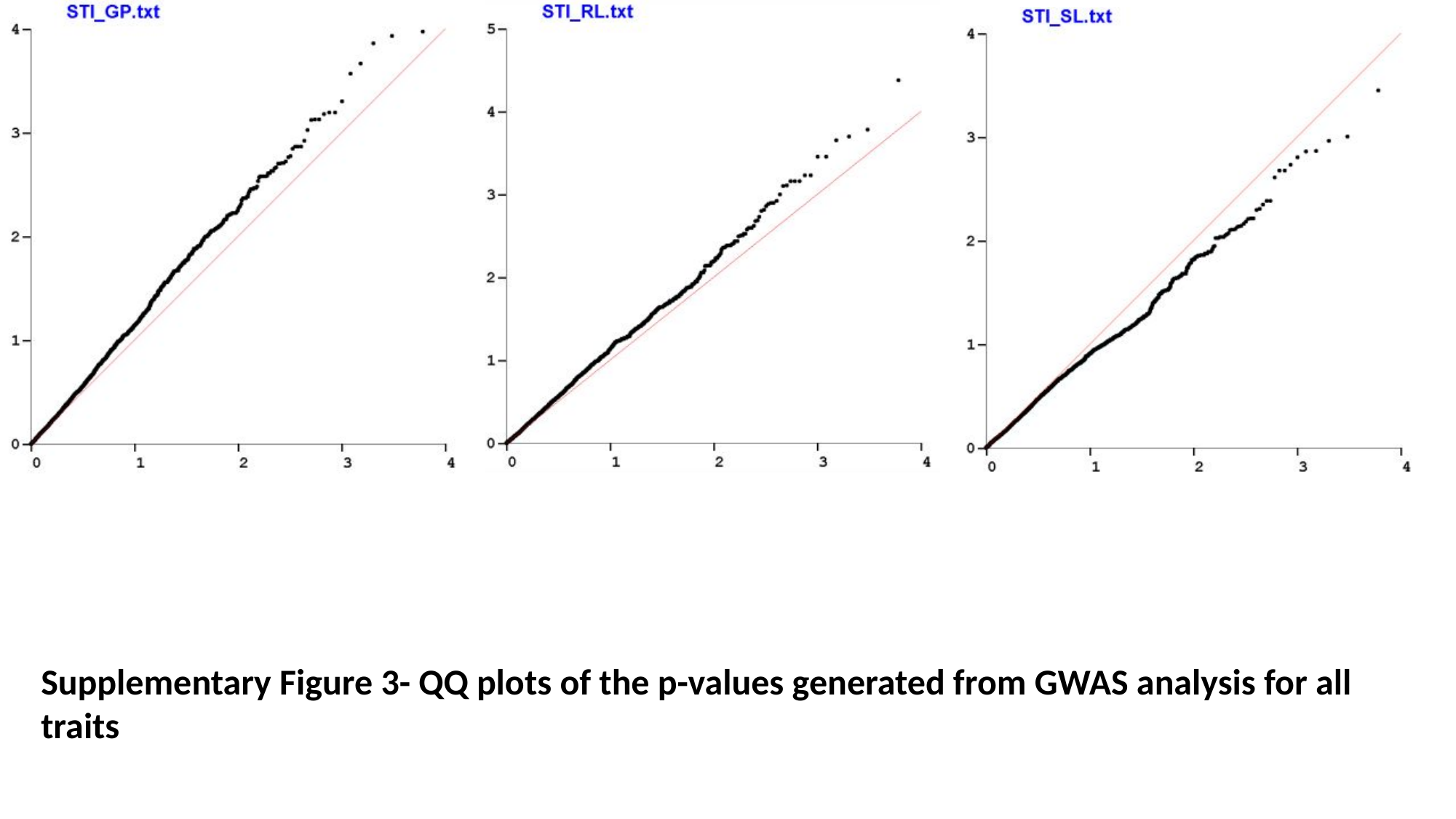

Supplementary Figure 3- QQ plots of the p-values generated from GWAS analysis for all traits
